# An efficient modified method for plant leaf lipid extraction results in improved recovery of phosphatidic acid

**DOI:** 10.1186/s13007-018-0282-y

**Published:** 2018-02-13

**Authors:** Sunitha Shiva, Regina Enninful, Mary R. Roth, Pamela Tamura, Krishna Jagadish, Ruth Welti

**Affiliations:** 1 Kansas Lipidomics Research Center, Division of Biology, Kansas State University, Manhattan, KS USA; 2 Department of Agronomy, Kansas State University, Manhattan, KS USA

**Keywords:** Lipid extraction, Lipidomics, Mass spectrometry, Arabidopsis, Sorghum

## Abstract

**Background:**

Lipidomics plays an important role in understanding plant adaptation to different stresses and improving our knowledge of the genes underlying lipid metabolism. Lipidomics involves lipid extraction, sample preparation, mass spectrometry analysis, and data interpretation. One of the practical challenges for large-scale lipidomics studies on plant leaves is the requirement of an efficient and rapid extraction method.

**Results:**

A single-extraction method with a polar solvent mixture gives results comparable to a widely used, multi-extraction method when tested on both *Arabidopsis thaliana* and *Sorghum bicolor* leaf tissue. This single-extraction method uses a mixture of 30 parts chloroform, 25 parts isopropanol, 41.5 parts methanol, and 3.5 parts water (v/v/v/v) and a 24-h extraction time. Neither inclusion of ammonium acetate nor inclusion of acetic acid increased extraction efficiency.

**Conclusions:**

The extract produced by this method can be used for analysis by mass spectrometry without a solvent evaporation step. The amount of lipid extracted, including phosphatidic acid, is comparable to widely used, more labor-intensive methods. The single-extraction protocol is less laborious, reducing the potential for human error.

**Electronic supplementary material:**

The online version of this article (10.1186/s13007-018-0282-y) contains supplementary material, which is available to authorized users.

## Background

**Fig. 1:**
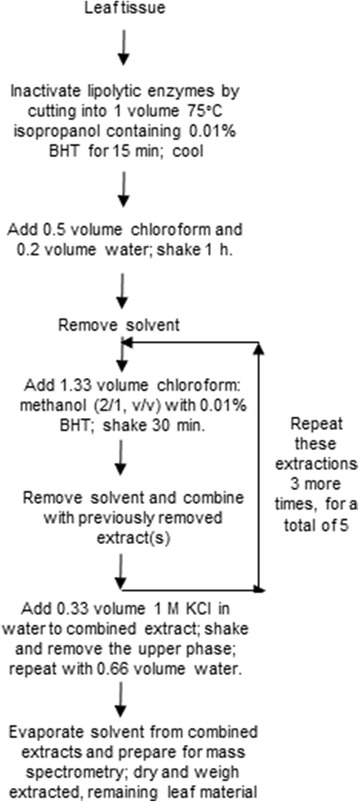
The Ryu and Wang extraction method [2], as modified by Welti et al. [1] and performed for Arabidopsis leaf extraction in the current work. This is a common extraction method for leaf lipidomics. Abbreviation not indicated previously: BHT, butylated hydroxytoluene

Lipidomics, typically using mass spectrometry to quantify lipids, requires simple, rapid, and efficient extraction methods for high sample throughput. An extraction method introduced for plant lipidomics by Welti et al. [1] was originally described by Ryu and Wang [2]. The Ryu and Wang method (Fig. 1) begins with an extraction using a solvent mixture similar to that used by Bligh and Dyer [3]. The Bligh and Dyer method uses a one-phase system with chloroform: methanol: water (1/2/0.8, v/v/v) for tissue extraction, with tissue water included in the water amount. In the Bligh and Dyer method, the extraction is followed by addition of more chloroform and methanol to make two phases, with the lipid in the chloroform phase. The method described by Ryu and Wang [2] incorporates a hot isopropanol treatment, designed to inhibit lipolytic enzymes, particularly phospholipase D, in plant tissues. This hot isopropanol treatment was originally used by de la Roche and Andrews [4] and also described by Moore [5]. Whereas the de la Roche et al. method involved two extractions with isopropanol followed by a standard Bligh and Dyer extraction, Moore [5] and Ryu and Wang [2] extracted by adding chloroform and water directly to the isopropanol in which plant tissue had been heated to form a single phase. The final solvent proportions were the same as in the Bligh and Dyer extraction, with the isopropanol substituting for the methanol. After shaking and removing the solvent, Ryu and Wang [2] re-extracted the leaf tissue twice with chloroform: methanol (2/1, v/v), and others have used additional re-extractions (e.g. [1]). The multiple extractions make the Ryu and Wang method laborious, and, hence, limit sample throughput. Still, this approach has been used widely for extracting various plant tissues, e.g., Arabidopsis leaves, roots, flowers, stems, and seeds [1, 6–12], soybean leaves and roots [13], wheat leaves [14, 15], rice leaves [12], and zoysiagrass rhizomes [16].

In order to analyze lipids by mass spectrometry in a high-throughput manner, a streamlined single-extraction method for Arabidopsis leaves was recently established by members of our laboratory [17]. After quenching the samples in hot isopropanol, a mixture of solvents, optimized for mass spectrometry with an electrospray ionization source, was added. The solvent mixture was chosen so that the extracted mixture could be infused directly into a mass spectrometer or, if it was too concentrated, simply diluted into the same solvent mixture and then infused, without a solvent evaporation step. The final solvent mixture was chloroform: isopropanol: methanol: 300 mM ammonium acetate in water (30/25/41.5/3.5, v/v/v/v). The procedure involved shaking the tissue with the solvent mixture at room temperature for 24 h. The ability of this solvent to extract each class was compared to the method described by Ryu and Wang [2] (also described by Welti et al. [1], with modifications). Among the lipid classes analyzed, monogalactosylmonoacylglycerol (MGMG), hexosylceramide (HexCer), and sterol derivatives extracted better by the Vu et al. method than by the method described by Ryu and Wang [2], whereas diacylglycerol (DAG) and phosphatidic acid (PA) extracted better by the Ryu and Wang method [17].

**Fig. 2:**
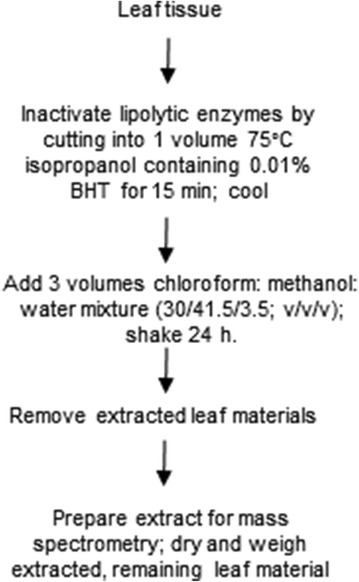
The single-extraction method tested in the current work for extraction of Arabidopsis and sorghum leaf materials

Overall, the Vu et al. method simplified sample preparation. However, the reduced extraction of some lipids, particularly the important signaling lipid and biosynthetic intermediate, PA, by the Vu et al. [17] method compared to the Ryu and Wang [2] method, was of some concern. We hypothesized that the ammonium acetate additive, which was included in the Vu et al. method to enhance ionization of lipids during their analysis by mass spectrometry, may have had a negative effect on the extraction of PA. Here, we demonstrate the effectiveness of a modification of the Vu et al. method, using chloroform: isopropanol: methanol: water (30/25/41.5/3.5, v/v/v/v), without additional additives, for extraction of leaf tissue from both dicot (*Arabidopsis thaliana*) and monocot (*Sorghum bicolor*) species (Fig. 2).

## Materials and methods

### Plant materials

Wild-type Arabidopsis (accession Columbia-0) plants were grown in a 72-well tray in soil in a growth chamber under 14/10-h day/night cycles at 21 °C with 60% humidity. Leaves of 5-week-old plants were utilized for the extraction test. A hemostat was used to wound leaves 5, 6, and 7 [18] of 20 plants across the mid-vein, as described by Vu et al. [17]. Wounded leaves were harvested 45 min after wounding.

A mature leaf of a *Sorghum bicolor* cultivar grown in a greenhouse was punched with a paper punch. Each sample contained three punches.

### Lipid extraction methods

#### Extraction materials

Glass tubes or vials with Teflon-lined caps were used in all methods. It is important to avoid most plastics when preparing samples for mass spectrometry. All solvents, including water, were HPLC-grade.

#### Ryu and Wang [2] extraction (modified Bligh and Dyer [3]) (Arabidopsis and sorghum)

Leaf materials (3 Arabidopsis leaves or 3 punches, with a paper punch, from a sorghum leaf) were harvested directly into a 50-ml tube containing 3 ml (Arabidopsis), or into a 4-ml vial containing 0.4 ml (sorghum), of isopropanol with 0.01% butylated hydroxytoluene (BHT). The isopropanol had been preheated to 75 °C. Each sample was incubated at 75 °C for 15 min and cooled to room temperature. Chloroform (1.5 ml for Arabidopsis; 0.2 ml for sorghum) and water (0.6 ml for Arabidopsis; 0.08 ml for sorghum) were added, and samples were shaken (1 h for Arabidopsis; 2 h for sorghum). Extracts were removed from the leaf material to new tubes. The leaf materials were re-extracted four times with 4 ml chloroform: methanol (2/1, v/v) with 0.01% BHT and 30 min of shaking each time (Arabidopsis), or three times over a 24-h period with 0.5 ml chloroform: methanol (2/1, v/v) with 0.01% BHT and shaking each time (sorghum). All extracts from one leaf or leaf punch sample were combined. The combined Arabidopsis extracts were washed with 1 ml of 1 M KCl and then with 2 ml of water. The combined extracts from each sample were evaporated and dissolved in 1 ml chloroform. Intact, extracted leaf material from each sample was transferred to a new vial using forceps, dried overnight at 105 °C, and weighed.

#### Single-extraction method with water (Arabidopsis and sorghum)

Leaf materials (3 Arabidopsis leaves or 3 punches from a sorghum leaf) were harvested directly into a 20-ml vial containing 4 ml isopropanol with 0.01% BHT (Arabidopsis), preheated to 75 °C, or for sorghum, into a 4-ml vial with 0.4 ml of the same preheated solvent. Each sample was incubated at 75 °C for 15 min and cooled to room temperature. A chloroform: methanol: water mixture (30/41.5/3.5, v/v/v; 12 ml for Arabidopsis; 1.2 ml for sorghum) was added, thus making a final solvent mixture with chloroform: isopropanol: methanol: water in the ratio 30/25/41.5/3.5 (v/v/v/v). Extracts were shaken at 100 rpm on an orbital shaker for 24 h. Intact, extracted leaf materials were transferred to a new vial using forceps, dried overnight at 105 °C, and weighed. Before lipid analysis, 280 µl of 600 mM ammonium acetate were added to the Arabidopsis samples. The final volume was 16.28 ml for Arabidopsis and 1.6 ml for sorghum. After the ammonium acetate addition, the overall ammonium acetate concentration (considering total solvent volume) in the Arabidopsis samples was 10.3 mM.

#### Single-extraction method with ammonium acetate [17] (Arabidopsis)

Leaf materials (3 Arabidopsis leaves) were harvested directly into a 20-ml vial containing 4 ml isopropanol with 0.01% BHT, preheated to 75 °C. Each sample was incubated at 75 °C for 15 min and cooled to room temperature. A chloroform: methanol: 300 mM ammonium acetate in water mixture (30/41.5/3.5, v/v/v; 12 ml) was added to make a final solvent mixture with chloroform: isopropanol: methanol: 300 mM ammonium acetate in water (30/25/41.5/3.5, v/v/v/v), and extracts were shaken at 100 rpm on an orbital shaker for 24 h. The final volume was 16 ml, and the overall ammonium acetate concentration was 10.5 mM. Intact, extracted leaf materials were transferred to a new vial using forceps, dried overnight at 105 °C, and weighed.

#### Single-extraction method with acetic acid (Arabidopsis)

Leaf materials (3 Arabidopsis leaves) were harvested directly into a 20-ml vial containing 4 ml isopropanol with 0.01% BHT, preheated to 75 °C. Each sample was incubated at 75 °C for 15 min and cooled to room temperature. A chloroform: methanol: 300 mM acetic acid in water mixture (30/41.5/3.5, v/v/v/v; 12 ml) was added to make a final solvent mixture with chloroform: isopropanol: methanol: 300 mM acetic acid in water (30/25/41.5/3.5, v/v/v/v), and extracts were shaken at 100 rpm on an orbital shaker for 24 h. Intact, extracted leaf materials were transferred to a new vial using forceps, dried overnight at 105 °C, and weighed. Before lipid analysis, 600 mM ammonium hydroxide (280 µl) was added to the Arabidopsis samples. The final volume was 16.28 ml. With the ammonium hydroxide addition to the acetic acid-containing solution, the overall ammonium acetate concentration in the samples was 10.3 mM.

#### Single-extraction method with water with a repeat extraction (sorghum)

Sorghum leaf punches, extracted by the single-extraction method with water (above), were re-extracted a second time with 1.2 ml of chloroform: methanol: water (30/66.5/3.5, v/v/v), immediately after the first extraction, with shaking for 48 h. The two extracts were combined (total volume of 2.8 ml) before the extracted leaf materials were transferred to a new vial using forceps, dried overnight at 105 °C, and weighed.

### Analysis of lipids by ESI-triple quadrupole MS

#### Multiple reaction monitoring method for analysis of Arabidopsis lipids

Quality control and analytical samples were prepared and analyzed as previously described by Vu et al. [17]. For each quality control and analytical sample, internal standards (Additional file 1: Table S1) in 20 µl and a volume of sample corresponding to 0.04 mg extracted leaf dry mass were added to a 2-ml vial. As described in the previous section, Arabidopsis lipid aliquots were in chloroform (Ryu and Wang method), which was subsequently evaporated, or in chloroform: isopropanol: methanol: 300 mM ammonium acetate in water (30/25/41.5/3.5, v/v/v/v) or a close approximation of this mixture (all other methods). The total volume was brought to 1.4 ml with the same mixture, chloroform: isopropanol: methanol: 300 mM ammonium acetate in water (30/25/41.5/3.5, v/v/v/v). A multiple reaction monitoring method, operating in direct infusion mode with an electrospray ionization source on a tandem quadrupole mass spectrometer (Xevo TQS, Waters Corporation, Milford, MA), was used to acquire the mass spectral data [17]. 400 μl from each sample were used to fill a 300-μl loop for infusion at 30 μl min^−1^. Infusion was performed twice, acquiring data in positive (13 functions) and negative (7 functions) modes separately. Data were acquired on lipid analytes and internal standard components from 0 to 15 min, including a wash-out period, with repeated and continuous cycling through every multiple reaction monitoring function as described by Vu et al. [17]. Parameters used in the analysis are presented in Additional file 1: Table S1 for the internal standards and in Additional file 2: Table S2 for the Arabidopsis leaf lipids targeted in this study.

#### Precursor (Prec) and neutral loss (NL) scanning for analysis of sorghum lipids

Samples were prepared by adding internal standards (Additional file 3: Table S3) and 20 µl (Ryu and Wang and single-extraction) or 40 µl (single-extraction with repeat extraction) of sample corresponding to 0.2 mg extracted leaf dry mass. Samples were brought to 1.4 ml by adding chloroform: methanol: 300 mM ammonium acetate in water (30/66.5/3.5, v/v/v) for mass spectrometric analysis. Analysis was performed on a triple quadrupole mass spectrometer with an electrospray ionization source (API 4000 QTRAP, Applied Biosystems, Foster City, CA) in direct infusion mode. Samples were introduced using an autosampler (LC Mini PAL, CTC Analytics AG, Zwingen, Switzerland) fitted with the required injection loop for the acquisition time and presented to the electrospray ionization needle at 30 µl/min. Samples were analyzed with neutral loss and precursor scans. Most instrument settings were as indicated by Xiao et al. [19], but scan-specific analytical parameters are listed in Additional file 4: Table S4.

#### Mass spectral data processing

**Fig. 3:**
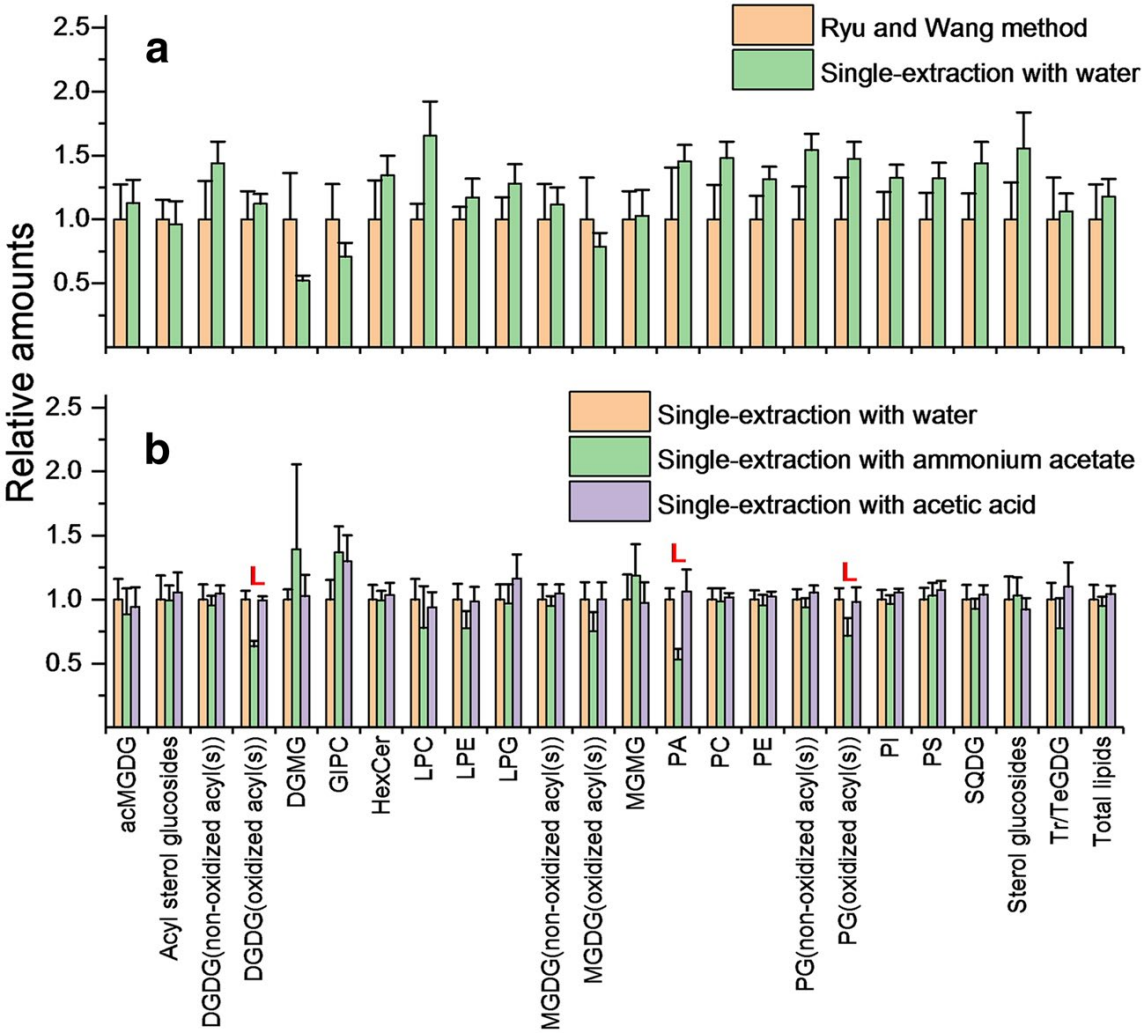
Method comparisons for lipid extraction of Arabidopsis leaves. Original data are presented in Additional file 5: Table S5. **a** Ryu and Wang method (Fig. 1; n = 4) is compared to single-extraction method with water (Fig. 2; n = 5). Ryu and Wang method was the reference method. Significance was determined by unpaired T test with unequal variance. There were no comparisons with adjusted p value < 0.05 with p value adjusted using the false discovery rate. **b** Addition of additives is compared to no additives in the single-extraction method with water (Fig. 2). The single-extraction method with water is the reference method. Significance was determined by one-way ANOVA with Tukey’s HSD test. Error bars indicate standard deviation. “L” indicates lipids whose levels were lower in the single-extraction with ammonium acetate method than in either of the other two methods (adjusted p value < 0.05). Abbreviations not indicated previously: acMGDG, acylated monogalactosyldiacylglycerol; DGDG, digalactosyldiacylglycerol; DGMG, digalactosylmonoacylglycerol; GIPC, glycosylinositolphosphoceramide; LPC, lysophosphatidylcholine; LPE, lysophosphatidylethanolamine; LPG, lysophosphatidylglycerol; MGDG, monogalactosyldiacylglycerol; PC, phosphatidylcholine; PE, phosphatidylethanolamine; PG, phosphatidylglycerol; PI, phosphatidylinositol; PS, phosphatidylserine; SQDG, sulfoquinovosyldiacylglycerol; Tr/TeGDG, tri- and tetra-galactosyldiacylglycerols

**Fig. 4:**
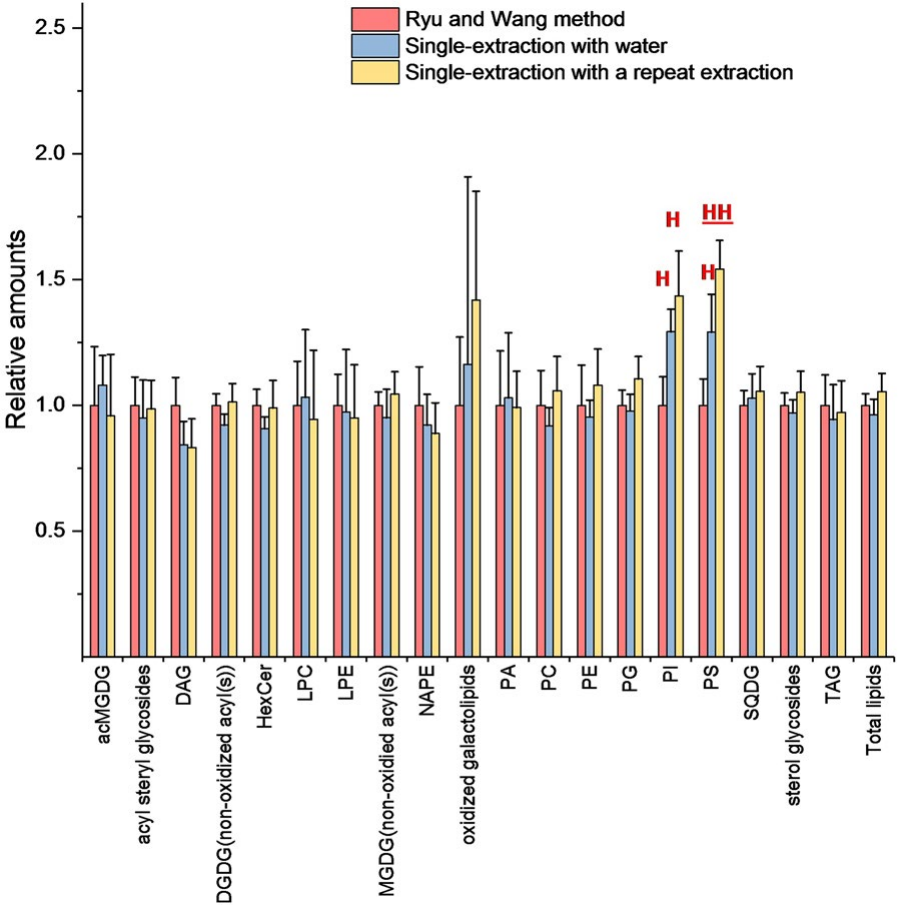
Method comparisons for lipid extraction of sorghum leaf punches. Original data are presented in Additional file 6: Table S6. Ryu and Wang method (Fig. 1; n = 7), as the reference method, compared to single-extraction method with water (Fig. 2; n = 7) and the single-extraction method with a second extraction (n = 7). Significance was determined by one-way ANOVA with Tukey’s HSD test. Error bars indicate standard deviation. “H” indicates PI and PS were higher in the indicated method than in the Ryu and Wang method and “HH” indicates that PS was higher in the single-extraction method with a repeat extraction than in either of the other methods (adjusted p < 0.05 with p value adjusted using the false discovery rate). Abbreviations not indicated previously: TAG, triacylglycerol, NAPE, N-acyl phosphatidylethanolamine

Multiple reaction monitoring data from the Waters Xevo TQS mass spectrometer were processed as described by Vu et al. [17]. Prec and NL data were processed as described by Xiao et al. [19], using LipidomeDB Data Calculation Environment (http://129.237.137.125:8080/Lipidomics/). In all cases, data were calculated as “normalized mass spectral signal” × (dried, extracted tissue mass)^−1^, with “normalized” indicating that the values were determined in relation to the intensities of the internal standards, as indicated in Additional file 1: Table S1, Additional file 2: Table S2, Additional file 3: Table S3, and Additional file 4: Table S4. In addition to processing of Prec and NL scans described by Xiao et al. [19], the monogalactosyldiacylglycerol (MGDG) and digalactosyldiacylglycerol (DGDG) data from the neutral loss mode were corrected by division by 2.8 because we determined that the responses of the unsaturated, plant-derived galactolipid species are 2.8 times greater than the internal standards, when measured in NL mode with [M + NH_4_]^+^ ions. Multiple reaction monitoring data are uncorrected for response factors. Coefficients of variation (CoV), shown in Additional file 5: Table S5 and Additional file 6: Table S6, were calculated from the quality control samples for the Arabidopsis data and from the samples extracted by the Ryu and Wang method for the sorghum data. Lipid analytes used to evaluate the extraction methods had CoV < 0.2 for Arabidopsis lipids and < 0.3 for sorghum lipids (Additional file 5: Table S5 and Additional file 6: Table S6). Finally, data shown in Figs. 3 and 4 were calculated by dividing all averages and standard deviations, for each lipid class, by the average of the reference dataset, as indicated in the figure legends, for that lipid class.

## Results and discussion

Recent advances in genomics provide new opportunities to link plant phenotypes to gene function. However, genome-wide association studies and other genetic linkage studies, such as those linking phenotypes with mutations, require hundreds or, in some cases, thousands of measurements of each plant phenotype (e.g., [20, 21]). If lipidomics is to be applied to discover new genetic functions for lipids, by linking levels of specific lipids with variation in specific genes, the extraction of lipids from plant tissues is a potentially rate-limiting step. Thus, there is a strong impetus to demonstrate a lipid extraction method that extracts as well as the major currently used approach, but which involves less effort. The approach described here utilizes extended shaking with a polar solvent in place of shorter, repeated extractions, which require considerably more labor.

An approach, involving the inactivation of lipases with isopropanol and the use of a polar mixture of chloroform, isopropanol, methanol, and ammonium acetate in water for a long (24 h) extraction, was developed in our laboratory by Vu et al. [17]. Importantly, the hot isopropanol treatment that minimizes lipolytic activity ([4, 5]) was incorporated into this single-extraction approach. Although satisfactory for most lipids, the long extraction with chloroform, isopropanol, methanol, and ammonium acetate in water (the “single-extraction with ammonium acetate”) did not extract PA, a major signaling lipid class, as well as the widely used Ryu and Wang method [2, 17]. Thus, we tested the hypothesis that deleting the ammonium acetate additive from the extraction mixture might make the method equivalent in extraction ability to the Ryu and Wang method. Figure 2 outlines the modified single-extraction method using a solvent mixture containing water without additives. To test the hypothesis, we analyzed samples extracted by both the methods shown in Figs. 1 and 2, measuring 163 lipid analytes from 23 classes using a targeted, multiple reaction monitoring mass spectrometry method, designed to optimize precision for sample comparison, rather than absolute quantification [17].

In Fig. 3a, we show that the modified method, with solvent mixture containing no added salt, extracts each of the measured lipid classes, including PA, without significant difference from the Ryu and Wang method [2]. In Fig. 3b, we compare three extraction methods, the single-extraction method with water, the Vu et al. [17] extraction method (the single-extraction with ammonium acetate), and a single-extraction method with acetic acid. The single-extraction with acetic acid was added to test whether acidification might provide extraction benefits. We found that there was no difference in the extraction efficiency between the method with water and the method with acetic acid. However, the Vu et al. [17] method, which uses the additive ammonium acetate, showed lower amounts of lipids, including PA, ox-DGDG, and ox-PG. The relatively poor extraction of PA was also observed by Vu et al. [17]. Hence, the modified single-extraction method with a solvent mixture containing water is an efficient method with high reproducibility for large-scale lipidomic studies. It should be noted that previous work demonstrated that glycosylinositolphosphoceramides (GIPC) are not quantitatively extracted with chloroform: methanol: water [22]. Indeed, if polyglycosphingolipids are the lipid classes of primary interest, the extraction method of Toledo et al. [23], as described by Markham et al. [22], is recommended.

We also tested the efficiency of this single-extraction method with water on leaf punches from the monocot, sorghum. Here, we measured a total of 118 lipid analytes from 19 classes using a targeted mass spectrometry approach using precursor and neutral loss scans to evaluate the extraction. We tested two hypotheses, that the single-extraction method with water would extract sorghum leaves as well as the Ryu and Wang method and that adding a second extraction would not improve the method. Figure 4 shows the single-extraction method with water and the “single-extraction method with a repeat extraction”, compared to the Ryu and Wang method. Statistical analysis shows that PI and PS extract slightly better in sorghum leaves with the single-extraction method with water than with the Ryu and Wang method, while all other lipid classes are not significantly different in amount extracted. Thus, contrary to our second hypothesis, there is a slight further increase in PS level by including an additional round of extraction. This might suggest that a second round of extraction would be warranted in circumstances where accurate quantification was desired. However, in our judgment, the single extraction approach with water is sufficient for large-scale comparative work.

Overall, the current data support the notion that the modified single-extraction method with water works as well or better than the widely used multi-step Ryu and Wang method [2], also used by Welti et al. [1]. The efficiency of this method is due to the use of a polar solvent mixture and an increase in the extraction time. This modified single-extraction also improves the extraction of PA compared to Vu et al. [17].

## Conclusion

The modified single-extraction method with water presented herein is an efficient lipid extraction approach comparable to widely used methods, and is suitable for large-scale lipidomics applications using leaves of model and crop plants. Poor extraction of the important signaling compound PA, as seen with the Vu et al. method, is improved with this newly modified approach. Extracts can be prepared for lipidomics without solvent evaporation. It is less laborious than traditional methods, and the shorter protocol is likely to reduce human error and facilitate mechanization of the extraction method and analytical sample preparation.

## Additional files

**Additional file 1.** Internal standards used in Arabidopsis lipid profiling using multiple reaction monitoring.

**Additional file 2.** Arabidopsis leaf lipid experimental parameters. Arabidopsis lipids were analyzed by multiple reaction monitoring on a Waters Xevo TQS mass spectrometer.

**Additional file 3.** Internal standards used in sorghum lipid profiling.

**Additional file 4.** Sorghum leaf lipid experimental parameters. Sorghum lipids were analyzed by precursor and neutral loss scanning on an Applied Biosytems 4000 QTRAP mass spectrometer.

**Additional file 5.** Arabidopsis thaliana leaf lipid experimental data from four extraction methods.

**Additional file 6.** Sorghum leaf lipid experimental data from three extraction methods.
